# The Cough Repertory

**Published:** 1883-11

**Authors:** 


### THE COUGH REPERTORY.

The first eight pages of this repertory have been re-arranged, and are given with this number. Also the pages completing part first. In next issue we propose to complete this work, and also Dr. Wells' article on Typhoid Fever.

